# Supplementary material for: Direct contribution of the sensory cortex to the judgment of stimulus duration
Source: Nat Commun. 2024 Feb 24;15:1712. doi: 10.1038/s41467-024-45970-0 (PMC10894222; doi:10.1038/s41467-024-45970-0)
Supplement: Supplementary file 1 — Supplementary Information [file 41467_2024_45970_MOESM1_ESM.pdf]

# Supplementary Information - **Direct contribution of the sensory cortex to the judgment of stimulus duration**

Reinartz S., Fassihi A., Ravera M., Paz L., Pulecchi F., Gigante M., Diamond M.E.

Supplementary figures:

**S1.** Stimulus generalization matrix.

**S2.** Control experiments test for effects of optogenetic intervention on duration judgment acuity.

**S3.** Psychometric curves of the duration rats under optogenetic vS1 excitation.

**S4.** Psychometric curve fitting parameters for vS1 photoexcitation and photoinhibition of all duration rats.

**S5.** Bias in duration perception generalizes to different experimental conditions.

**S6.** Intensity-evoked bias in duration perception under different optogenetic conditions.

**S7.** Neuronal responses in vS1 to vibrissal stimulation and photoexcitation in rats trained for duration discrimination (n = 5).

**S8.** Neuronal responses in vS1 to vibrissal stimulation and optogenetic excitation in rats trained for intensity discrimination (n = 3).

**S9.** vS1 intensity coding.

**S10.** Quantification of vS1 intensity coding.

**S11.** vS1 duration coding.

**S12.** Gaussian mixture model (GMM) of vS1 neuronal activity.

**S13.** Single vS1 neuron responses can be simulated by fitting input current parameters; classification of vS1 neurons.

**S14.** Congruent and incongruent stimulus pairs reveal interacting perception of duration and intensity.

Supplementary tables:

**T1.** Model fitted parameters.

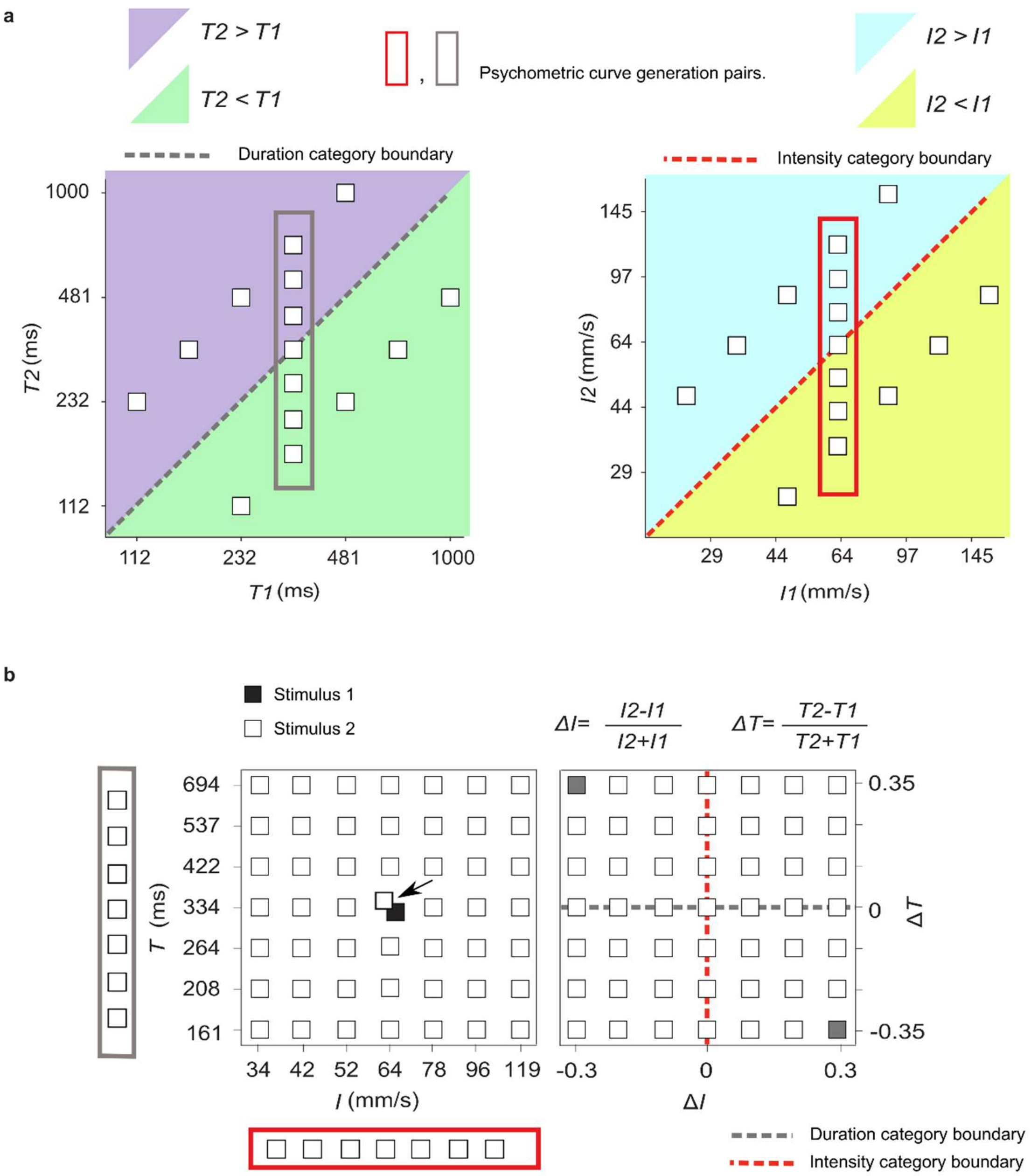


**Supplementary Figure 1. Stimulus generalization matrix. a.** The entire stimulus set was used for both duration and intensity rats. *T1* and *T2* duration combinations are represented by blank squares (left panel). The diagonal gray dashed line indicates the category boundary (*T2* > *T1* vs *T2* < *T1*). The right panel accordingly displays possible *I1* and *I2* values that are relevant to the intensity task. For both duration and intensity rats, all possible combinations of [*T1*, *T2*] and [*I2*, *I1*] were presented randomly across trials. The [*T1*, *T2*] and [*I2*, *I1*] combinations inside the gray and red rectangle are trials that were used to generate psychometric curves. **b.** Stimulus combinations that were used to generate psychometric curves in order to assess bias and acuity. Left panel: trials in which *T2* and *I2* (empty squares) varied in small steps, while *T1* and *I1* (central filled square) were fixed (334 ms, 64 mm/s). Right panel illustrates the normalized duration and intensity differences, corresponding to the stimulus pairs in the left panel. Duration rats must base their choice on Δ*T* values (above and below the gray dashed line), while intensity rats must choose based on Δ*I* values (left and right side of the red dashed line). Gray filled squares represent stimulus pairs illustrated in Fig. 1a example stimulus traces.

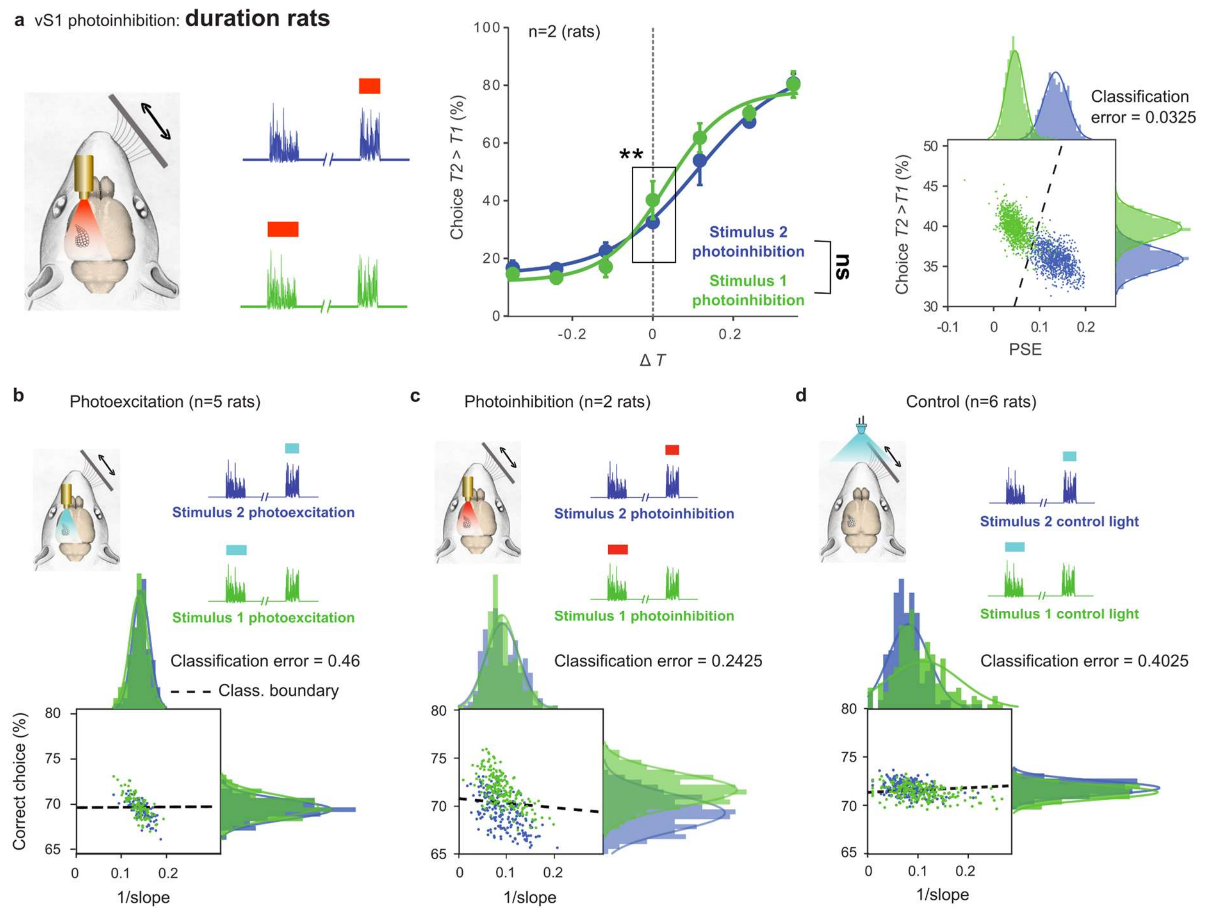


**Supplementary Figure 2. Control experiments test for effects of optogenetic intervention on duration judgment acuity. a.** Left: Photoinhibition during either vibrissal stimulus 2 or stimulus 1. Middle: Psychometric curves derived from stimulus 2 photoinhibition (blue) or stimulus 1 photoinhibition (green). SEM across individual rats indicated as error bars. To uncover optogenetic effects when not overridden by strong sensory evidence, statistical significance was tested on the stimulus pairs where $\Delta T = 0$ (permutation test, 1000x; $p = 0.009$). When evaluating statistical significance on all pairs (excluding the "easy") as in Fig2b-e (middle), no significant difference ($p > 0.1$) was found between the two conditions. Right: two signatures of a possible curve shift – percent of trials judged as *T2 > T1* irrespective of stimulus duration (ordinate) and PSE (abscissa) – were measured with bootstrap resampling (1000x). A support vector machine classifier quantified data separation by classification error. **B.** Effect of photoexcitation on duration judgment acuity. Two signatures of the acuity, percent correct (ordinate) and the psychometric curve's inverse slope (abscissa), were measured with bootstrap resampling. A support vector machine classifier quantified the separation in the data. Light-on during the stimulus 2 (blue) did not significantly alter acuity as compared to light-on during stimulus 1 (green). **C.** and **d.** are same as **b**, but for photoinhibition and control sessions, respectively. Source data are provided as a Source Data file.

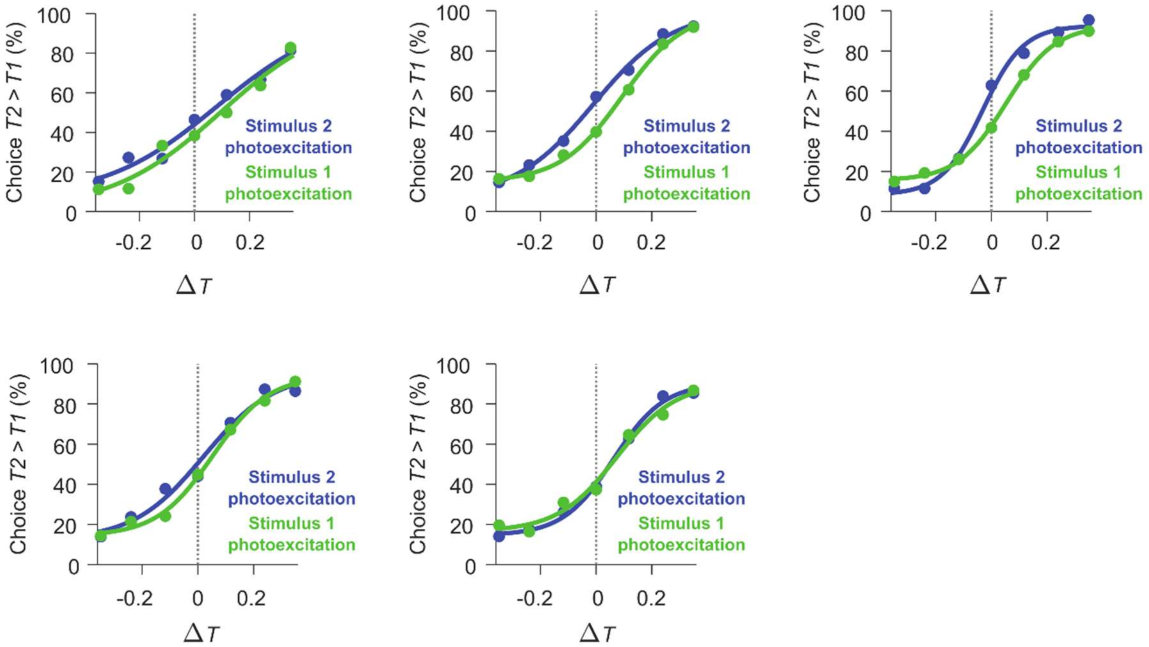


**Supplementary Figure 3. Psychometric curves of the *duration* rats under optogenetic vS1 excitation.** Photoexcitation of left vS1 during vibrissal stimulation leads to a bias in most rats. Photoexcitation during stimulus 2 (blue); photoexcitation during stimulus 1 (green). Source data are provided as a Source Data file.

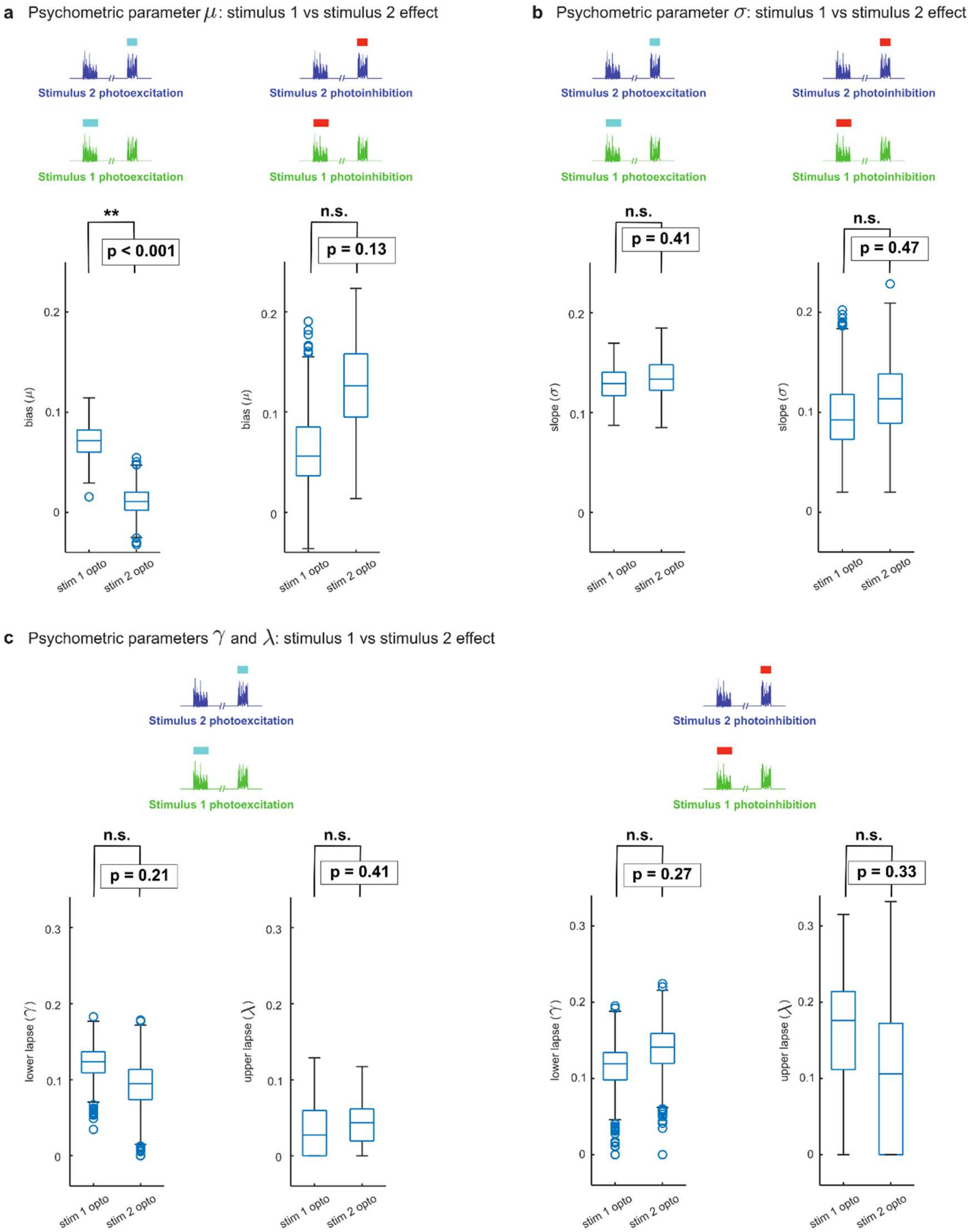


**Supplementary Figure 4. Psychometric curve fitting parameters for vS1 photoexcitation and photoinhibition of all duration rats. A.** Comparison of the shift in the bias parameter ($\mu$) between stimulus 1 versus stimulus 2 photoexcitation (left) and inhibition (right). **B.** Comparison of the shift in the slope parameter ($\sigma$) between stimulus 1 versus stimulus 2 photoexcitation (left) and photoinhibition (right). **C.** Comparison of the shift in the lapse parameters ($\gamma$, $\lambda$) between stimulus 1 versus stimulus 2 photoexcitation (left) and photoinhibition (right). Box plots of all 4 psychometric curve fitting parameters and their variability over resampling (1000x). The central line is the sample median, the edges of the box are the 25$^{th}$ and 75$^{th}$ percentiles, the whiskers extend to the most extreme data points that are no outliers, and the outliers are plotted as circles. Source data are provided as a Source Data file.

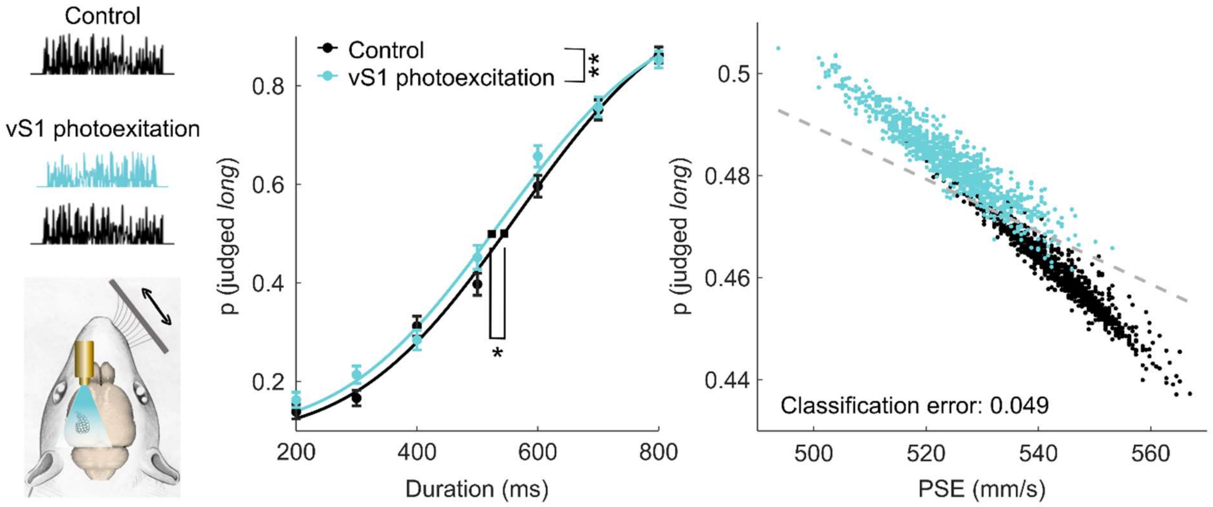


**Supplementary Figure 5. Bias in duration perception generalizes to different experimental conditions.** Four rats were trained in a reference memory (RM) task where, unlike the working memory task in the body of the paper, the single tactile stimulus presented in each trial had to be judged as shorter or longer than a fixed duration, 500 ms. Also differently from the main experiments, light delivery was stochastic. Left: photoexcitation of left vS1 with light that varied stochastically in amplitude during vibrissal stimulation. Middle: photoexcitation (light blue) yielded a duration overestimation in comparison to the no-light control trials (black). Psychometric curves (n = 4 rats) with squares corresponding to estimated points of subjective equality (PSE). Averaged psychometric data ($p$ = 0.0084, resampling method 1000x, permutation test) as well as PSEs of the fitted psychometric curves ($p$ = 0.0482, resampling method 1000x, permutation test) uncovered a statistically significant difference. Right: two signatures of the curve shift, percent of trials judged as *T2* > *T1* irrespective of stimulus duration (ordinate) and PSE (abscissa), were measured with bootstrap resampling (1000x). A support vector machine classifier quantifies data separation by classification error. Source data are provided as a Source Data file.

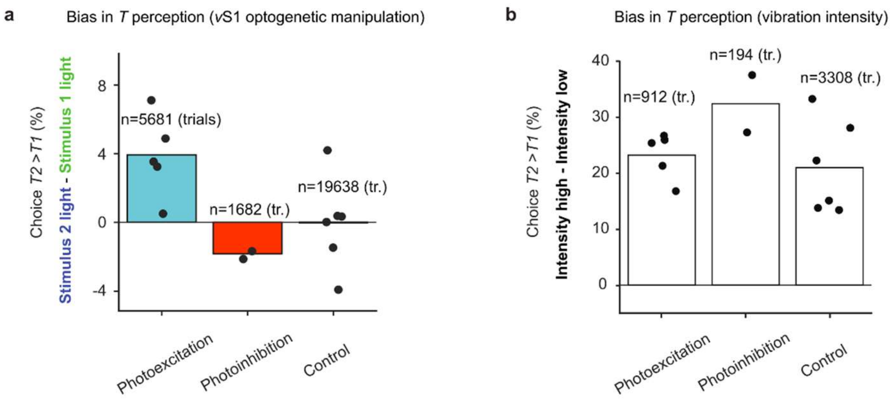


**Supplementary Figure 6. Intensity-evoked bias in duration perception under different optogenetic conditions. A.** Bias in duration perception induced by optogenetic manipulation. In the control experiments, a visible external light (465 nm) was applied during stimulus 1 or stimulus 2, with the same timing and occurrence as the light for optogenetic manipulations. Each dot represents a single subject. Bias quantified as the difference in percent of choices $T2 > T1$ averaged across all data points, excluding trials where $\Delta T = 0.35$ or -0.35 (for details, see Methods). Each dot represents a single subject, the bars illustrate the mean. **B.** Bias in duration perception evoked by high- versus low-intensity vibration (160 mm/s versus 64 mm/s, respectively) assessed under photoexcitation, photoinhibition, and the control no-light condition. The difference in choice $T2 > T1$ is measured by the method of Fig. 1d. Each dot represents a single subject; bars illustrate the mean. Intensity bias ($T2 > T1$ choice more likely when $I2 > I1$) was significant across all different experimental conditions (resampling method: permutation test 1000x, $p < 0.05$ for all three conditions). Photoexcitation, $p < 0.001$; photoinhibition, $p < 0.02$; control, $p < 0.001$. Source data are provided as a Source Data file.

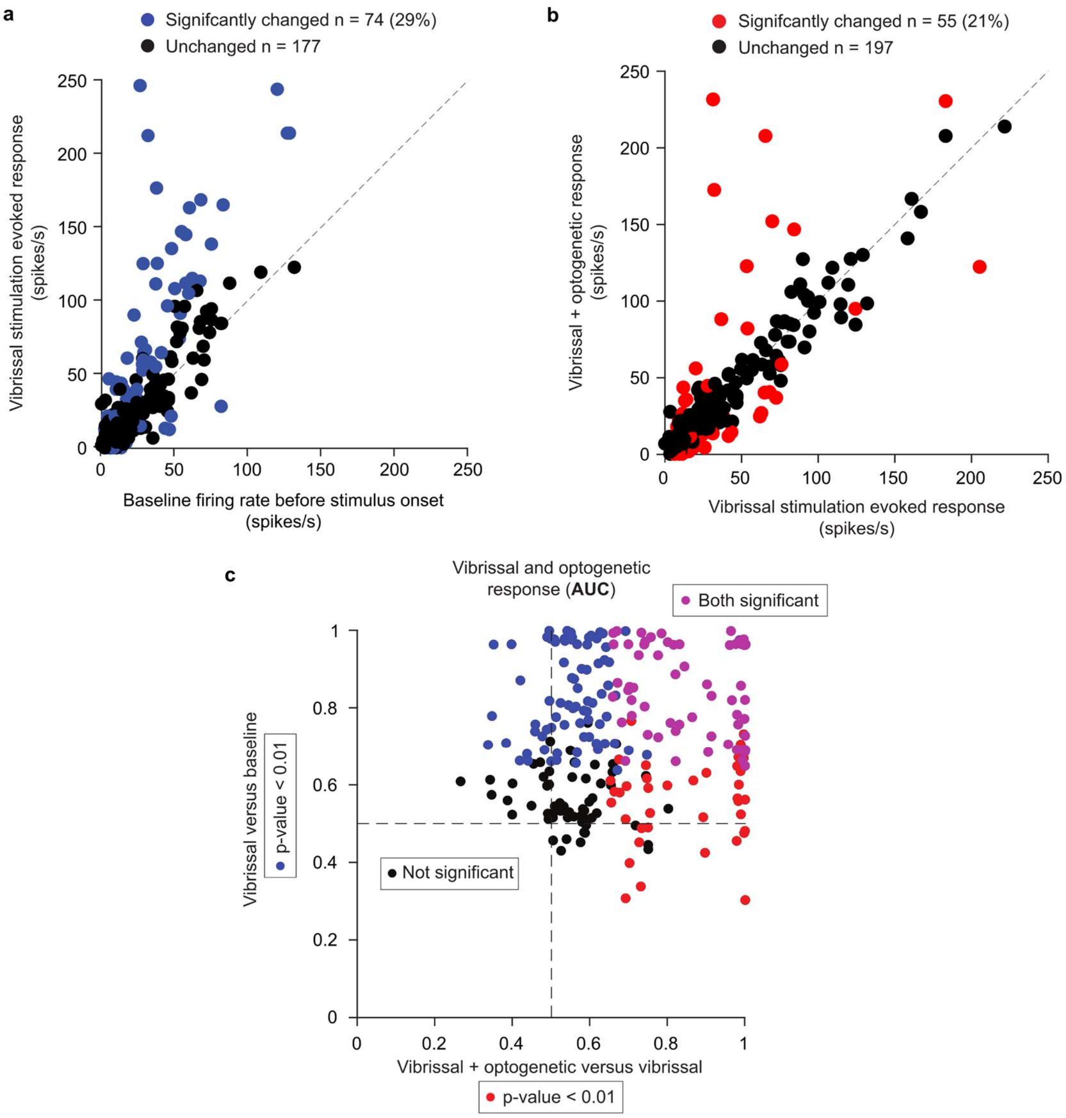


**Supplementary Figure 7. Neuronal responses in vS1 to vibrissal stimulation and photoexcitation in rats trained for duration discrimination (n = 5). A.** Average firing rate of individual vS1 units before stimulus onset (200 ms time window) versus the first 200 ms during vibrissal stimulation. Blue dots are neurons with significantly altered firing ($p < 0.01$, resampling method: permutation test, 500x) in response to vibrissal stimulation compared to background activity. **B.** Firing of all vS1 units is represented for the first 200 ms of vibrissal stimulation (abscissa), compared to the first 200 ms of vibrissal stimulation accompanied by photoexcitation (ordinate). Red dots show neurons with significantly increased and decreased firing rate ($p < 0.01$) during photoexcitation plus vibrissal stimulation as compared to vibrissal stimulation alone. **C.** Area under the ROC curve (AUC) was measured by comparing the response of neurons during the background activity versus vibrissal stimulation (ordinate). Additionally, the response during the vibrissal stimulation is compared to photoexcitation plus vibrissal stimulation (abscissa). Blue and red dots are neurons with significant response AUC for vibrissal stimulation only versus background activity and vibrissal stimulation versus photoexcitation plus vibrissal stimulation, respectively. Purple dots represent neurons with significant AUC for both comparisons. Significance was tested by the resampling method (permutation test, 500x). Source data are provided as a Source Data file.

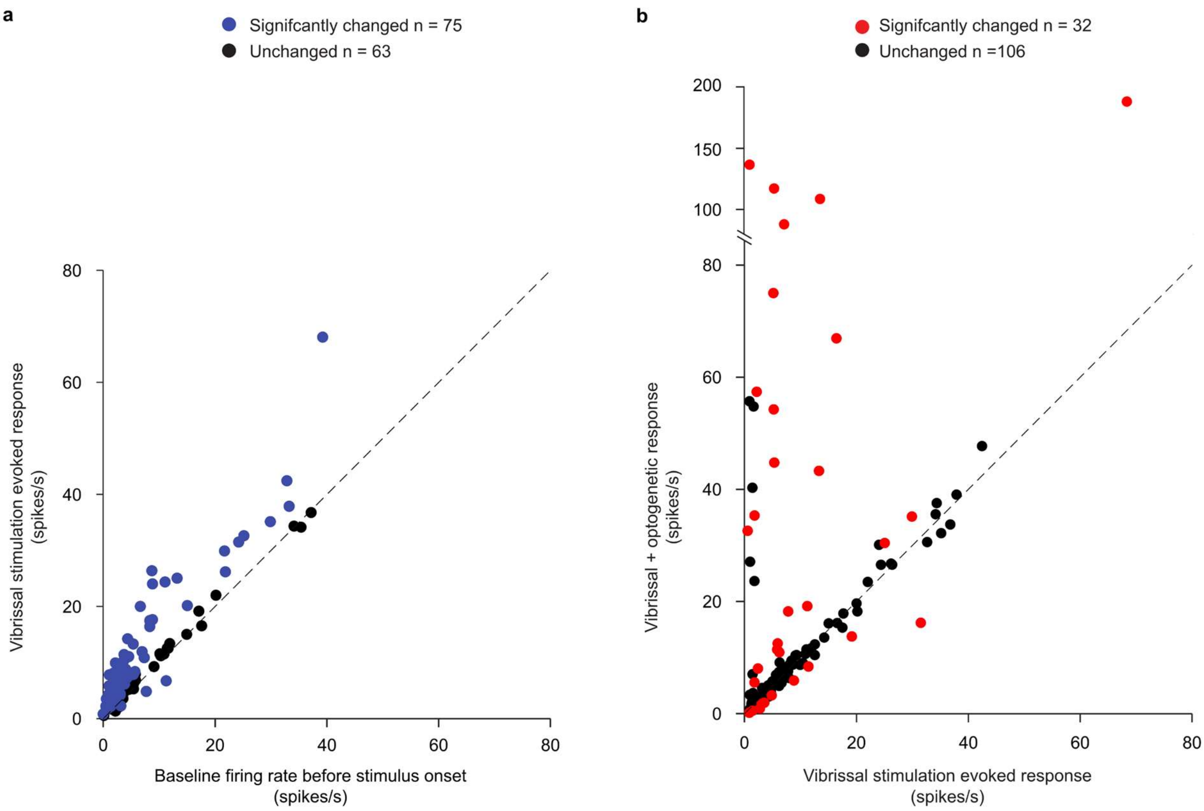


**Supplementary Figure 8. Neuronal responses in vS1 to vibrissal stimulation and optogenetic excitation in rats trained for intensity discrimination (n=3). A.** Average firing rate of individual vS1 units before stimulus onset (200 ms time window) versus the first 200 ms of vibrissal stimulation. Blue dots are neurons with significant vibrissal sensory response ($p < 0.01$, resampling method: permutation test, 500x) compared to background activity. **B.** Responses of all vS1 units for the first 200 ms of vibrissal stimulation (abscissa) versus the first 200 ms of vibrissal stimulation accompanied by photoexcitation (ordinate). Red dots show neurons with significantly increased and decreased firing rate ($p < 0.01$, resampling method: permutation test, 500x) during photoexcitation plus vibrissal stimulation as compared to vibrissal stimulation alone. Source data are provided as a Source Data file.

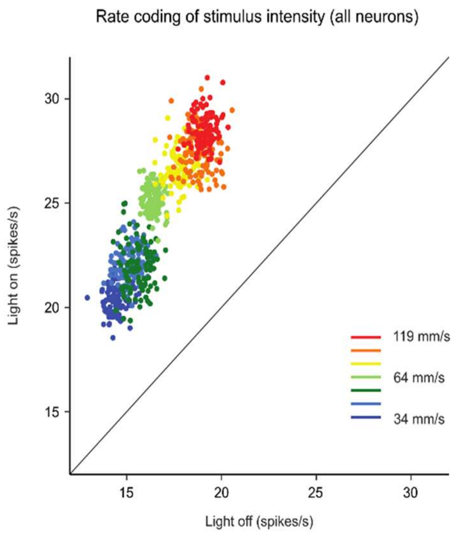


**Supplementary Figure 9. vS1 intensity coding.** Each dot shows population mean firing rate (bootstrap resampling) colored by stimulus intensity, recorded in EYFP-ChR2(H134R)-expressing rats (n = 3) performing the intensity task. The resampling algorithm (90% coding and 10% non-coding neurons [13]) was not applied; instead, all recorded neuronal clusters (n = 138) are included. Of these, 39 were "coding" neurons, showing a statistically significant correlation between between vibration intensity and firing rate ($p < 0.05$, resampling method: permutation test, 500x) and 99 were "non-coding" neurons, failing to show a statistically significant correlation between between vibration intensity and firing rate ($p > 0.05$). Source data are provided as a Source Data file.

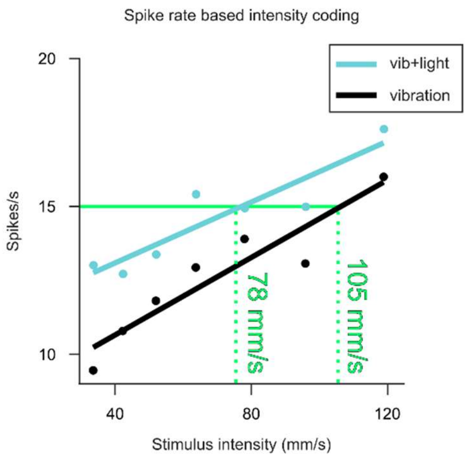


**Supplementary Figure 10. Quantification of vS1 intensity coding.** Each dot shows population mean firing rate for a given stimulus intensity, recorded in rats (n=3) performing the intensity task, with photoexcitation on (light blue) and off (black). Data obtained by population response resampling (see Methods). To reach a vS1 firing rate of 15 spikes/s (green horizontal line) a vibrissal vibration of mean speed 105 mm/s would be required with light-off; the same firing rate would be reached with vibration of mean speed 78 mm/s with light-on. Thus, as a first approximation optogenetic excitation would cause the 78 mm/s stimulus to establish a cortical firing rate representation normally evoked by a stimulus of 35% greater amplitude. The difference between the linear firing rate fits under the light-on (light blue) and light-off (black) conditions suggest that similar measures of optogenetic-induced misperception of vibration intensity could be made throughout the stimulus scale. Source data are provided as a Source Data file.

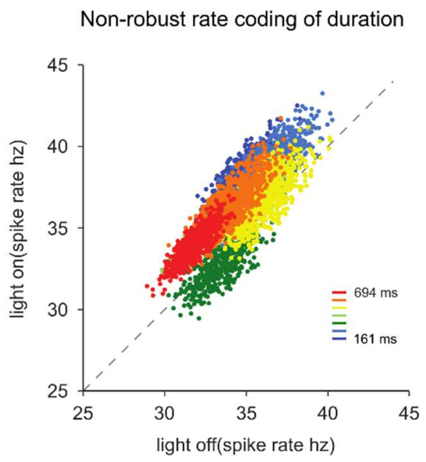


**Supplementary Figure 11. vS1 duration coding.** Each dot shows population mean firing rate (bootstrap resampling) across each respective stimulus duration (see color legend), recorded in EYFP-ChR2(H134R)-expressing rats (n = 5) performing the duration task. Source data are provided as a Source Data file.

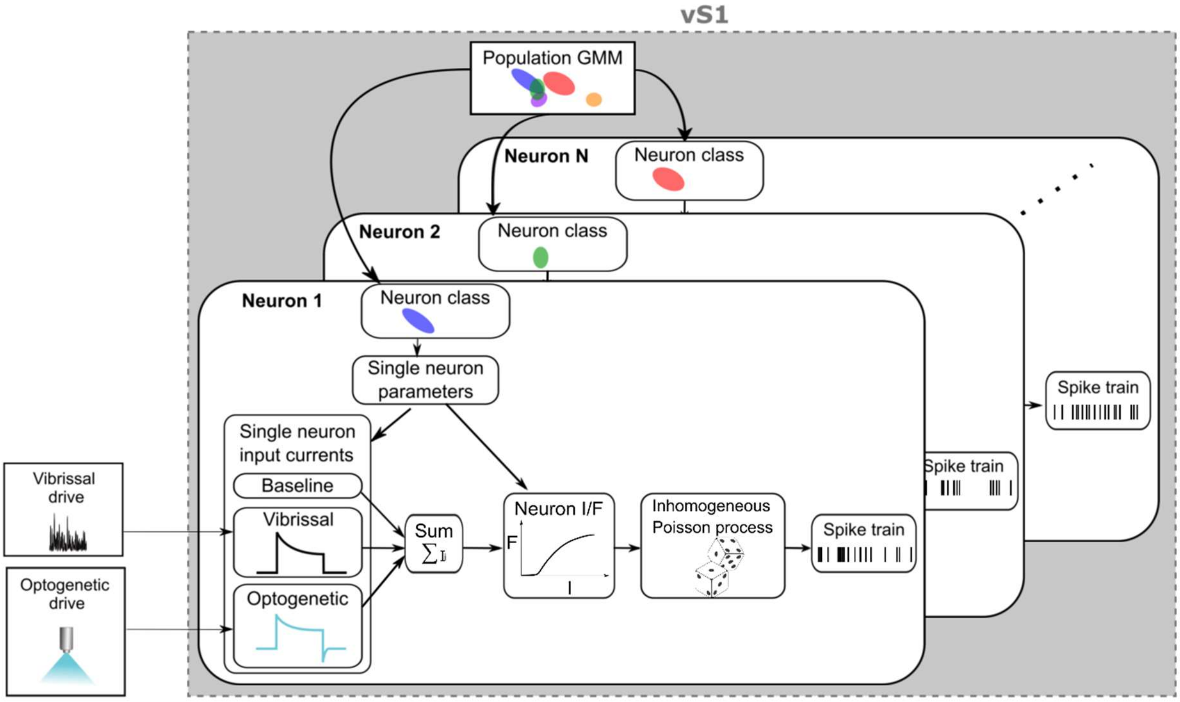


**Supplementary Figure 12. Gaussian mixture model (GMM) of vS1 neuronal activity.** Single vS1 spike trains in response to vibrissal stimulation and photoexcitation (see leftmost boxes) could be simulated by applying dynamic input currents to a Poisson process through a sigmoidal IF-curve (Eq. 10). The parameter set (see Eqs. 6-8) that determines the input currents and its dynamics, depending on vibrissal and optogenetic drive, was fitted to each recorded neuron individually. The distributions of this parameter set across all fitted neurons did not reveal distinct response-specific clusters and served as representative vS1 population characteristics. The GMM estimated a Gaussian distribution for each of the given parameters from the parameter values, given by fitting each individual neuron. Applying the GMM, representative vS1 model neurons could be resampled (see e.g., *Neuron 1* box), by retrieving a given input current parameter set from the parameters Gaussian distributions. Each model neuron consisted of a distinctive parameter set, representative to the recorded vS1 population that determined the input current, depending on vibrissal and optogenetic drive. Poisson spike trains of resampled model neurons were generated to simulate a vS1 population response (gray box) to a given external input.

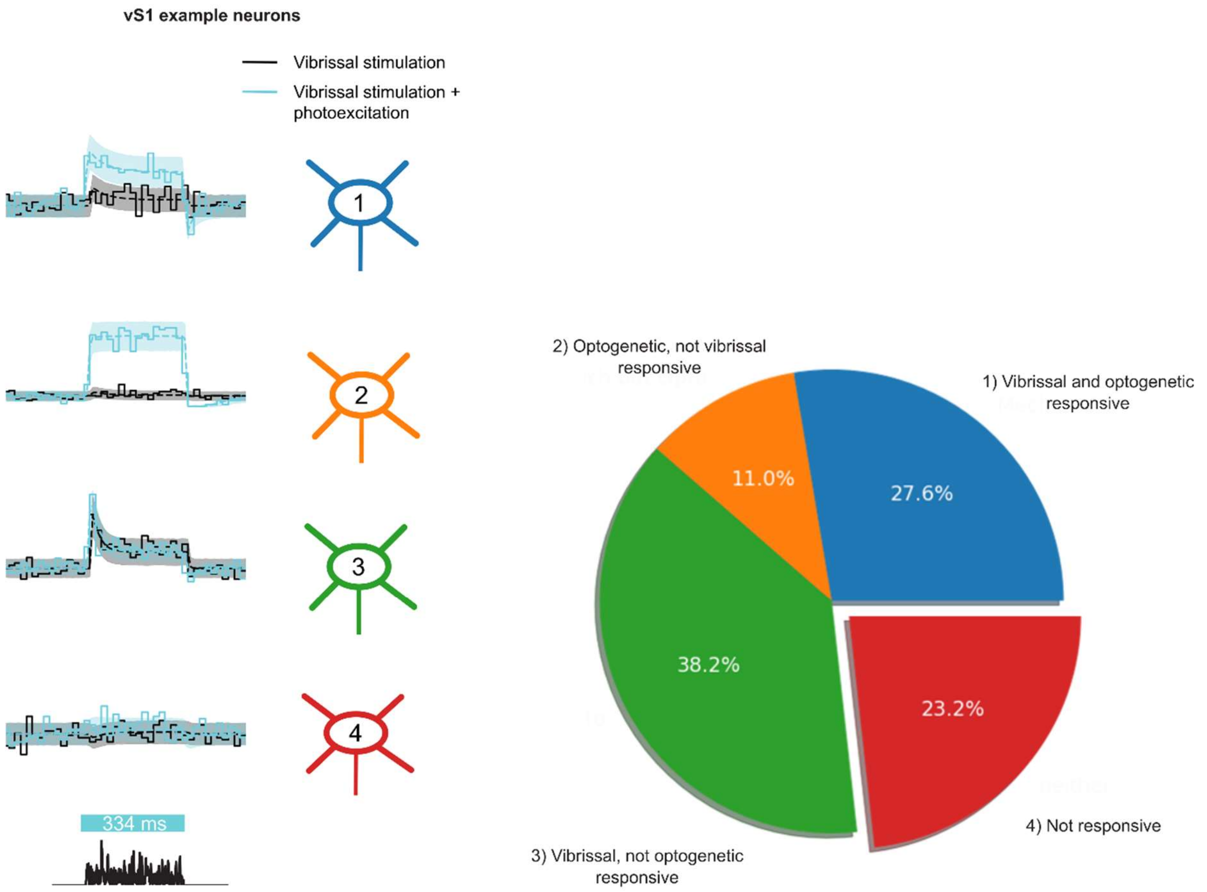


**Supplementary Figure 13. Single vS1 neuron responses can be simulated by fitting input current parameters; classification of vS1 neurons.** Left: PSTHs of four example neurons, responding to a 334 ms vibrissal stimulation (black, solid lines) and vibrissal stimulation plus photoexcitation (light blue, solid lines). Neuron-specific response characteristics, based on vibrissal and optogenetic drive, was simulated (dashed lines) by fitting a parameter set (see Eqs. 6-8) that determines the input currents for generating Poisson spike trains. Confidence intervals (shaded area) of the simulated PSTHs covered the variability of the data. Right: Assessing the fitted parameters for significant ($p < 0.005$) deviation from zero for vibrissal and optogenetic driven currents, allowed a neuron classification based on response properties (pie chart). Source data are provided as a Source Data file.

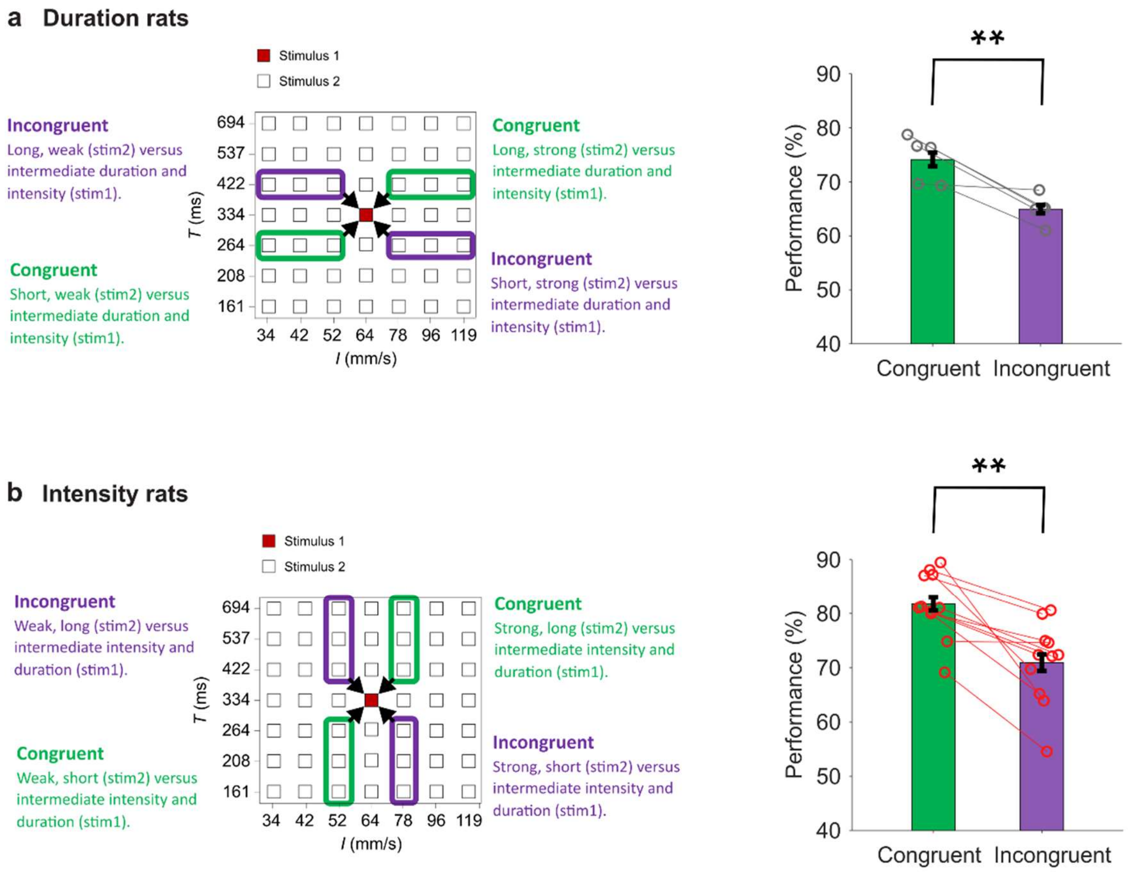


**Supplementary Figure 14. Congruent and incongruent stimulus pairs reveal interacting perception of duration and intensity. a.** Left panel: from the entire stimulus set presented to Duration rats, we consider pairs where the two features of stimulus 2 were either congruent (green) or incongruent (violet). In psychophysical experiments, rats compared all such instances of stimulus 2 to stimulus 1 (red box), of intermediate duration and intensity. Right panel: Trials from all Duration rats were pooled, and performance was seen to be significantly better (resampling method, permutation test, 1000x; $p < 0.001$) when congruent stimulus 2 was compared to stimulus 1 than when incongruent stimulus 2 was compared to stimulus 1. **b.** Left panel: In an analogous way, for Intensity rats we consider pairs where the two features of stimulus 2 were either congruent (green) or incongruent (violet). In psychophysical experiments, rats compared all such instances of stimulus 2 to stimulus 1 (red box), of intermediate intensity and duration. Right panel: Trials from all Intensity rats were pooled, and performance was seen to be significantly better (resampling method, permutation test, 1000x; $p < 0.001$) when congruent stimulus 2 was compared to stimulus 1 than when incongruent stimulus 2 was compared to stimulus 1. Source data are provided as a Source Data file.

| | | |
|---|---|---|
| $\tau$ | Leaky integration time constant | 990 ms |
| $\mu_b$ | Mean drive excluding vS1 | 1.95 |
| $\sigma_b^2$ | Variance of drive excluding vS1 | 208 |
| $p_L$ | Probability of lapse trial | 0.17 |
| $b_L$ | Probability of choosing *T2* > *T1* in lapse trial | 0.514 |

**Supplementary Table 1. Model fitted parameters.** Source data are provided as a Source Data file.
