# Supplementary material for: Direct contribution of the sensory cortex to the judgment of stimulus duration
Source: Nat Commun. 2024 Feb 24;15:1712. doi: 10.1038/s41467-024-45970-0 (PMC10894222; doi:10.1038/s41467-024-45970-0)
Supplement: Supplementary file 3 — Reporting Summary [file 41467_2024_45970_MOESM3_ESM.pdf]

nature portfolio

Corresponding author(s): Mathew E. Diamond

Last updated by author(s): Jan 23, 2024

Reporting Summary

Nature Portfolio wishes to improve the reproducibility of the work that we publish. This form provides structure for consistency and transparency in reporting. For further information on Nature Portfolio policies, see our Editorial Policies and the Editorial Policy Checklist.

Statistics

For all statistical analyses, confirm that the following items are present in the figure legend, table legend, main text, or Methods section.

n/a	Confirmed	
☐	☒	The exact sample size (*n*) for each experimental group/condition, given as a discrete number and unit of measurement
☐	☒	A statement on whether measurements were taken from distinct samples or whether the same sample was measured repeatedly
☐	☒	The statistical test(s) used AND whether they are one- or two-sided<br>*Only common tests should be described solely by name; describe more complex techniques in the Methods section.*
☐	☒	A description of all covariates tested
☐	☒	A description of any assumptions or corrections, such as tests of normality and adjustment for multiple comparisons
☐	☒	A full description of the statistical parameters including central tendency (e.g. means) or other basic estimates (e.g. regression coefficient) AND variation (e.g. standard deviation) or associated estimates of uncertainty (e.g. confidence intervals)
☐	☒	For null hypothesis testing, the test statistic (e.g. F, t, r) with confidence intervals, effect sizes, degrees of freedom and P value noted<br>*Give P values as exact values whenever suitable.*
☐	☒	For Bayesian analysis, information on the choice of priors and Markov chain Monte Carlo settings
☒	☐	For hierarchical and complex designs, identification of the appropriate level for tests and full reporting of outcomes
☐	☒	Estimates of effect sizes (e.g. Cohen's *d*, Pearson's *r*), indicating how they were calculated

Our web collection on statistics for biologists contains articles on many of the points above.

Software and code

Policy information about availability of computer code

Data collection	LabView, TDT data acquisition software
Data analysis	MATLAB

For manuscripts utilizing custom algorithms or software that are central to the research but not yet described in published literature, software must be made available to editors and reviewers. We strongly encourage code deposition in a community repository (e.g. GitHub). See the Nature Portfolio guidelines for submitting code & software for further information.

Data

Policy information about availability of data

All manuscripts must include a data availability statement. This statement should provide the following information, where applicable:

- Accession codes, unique identifiers, or web links for publicly available datasets
- A description of any restrictions on data availability
- For clinical datasets or third party data, please ensure that the statement adheres to our policy

The data that support the findings of this study are deposited to a public data repository and can be accessed through DOI: https://doi.org/10.5281/zenodo.1054878862. The data set includes rat behavioral data, neuronal data recorded in vS1 of rats that perform vibration intensity or duration discrimination, as well including optogenetic manipulation. Any additional information will be available from the authors upon reasonable request. Source data are provided with this paper.

Human research participants

Policy information about studies involving human research participants and Sex and Gender in Research.

Reporting on sex and gender	N/A
Population characteristics	N/A
Recruitment	N/A
Ethics oversight	N/A

Note that full information on the approval of the study protocol must also be provided in the manuscript.

Field-specific reporting

Please select the one below that is the best fit for your research. If you are not sure, read the appropriate sections before making your selection.

☒ Life sciences ☐ Behavioural & social sciences ☐ Ecological, evolutionary & environmental sciences

For a reference copy of the document with all sections, see nature.com/documents/nr-reporting-summary-flat.pdf

Life sciences study design

All studies must disclose on these points even when the disclosure is negative.

Sample size	20 male Wistar rats. We used multiple recordings and multiple animals to provide sufficient sample size and reported the sample sizes for all reported effects and statistical tests. We determined our sample size based on previous studies, but did not perform sample size calculation.
Data exclusions	All rats trained in that project were included. For optogenetics and electrophysiological recordings, all successfully implanted rats that recovered after the surgery and reached high performance in consecutive behavioral/neuronal recording sessions (n>=10) were included.
Replication	The experiments were replicated with a sample size to show statistical significance. About 200,000 trials from a total of 20 Wistar rats were independently tested.
Randomization	We randomized trials and test conditions in all presented analysis and experiments. Cross-validation was performed in order to test that no wrong conclusions were made due to limited sample size.
Blinding	Behavioral experiments, combining psychophysics, optogenetics and neuronal recordings were done in an automated setup controlled by custom made hardware and software independent to the conditions tested.

Reporting for specific materials, systems and methods

We require information from authors about some types of materials, experimental systems and methods used in many studies. Here, indicate whether each material, system or method listed is relevant to your study. If you are not sure if a list item applies to your research, read the appropriate section before selecting a response.

Materials & experimental systems

n/a	Involved in the study
☐	☒ Antibodies
☒	☐ Eukaryotic cell lines
☒	☐ Palaeontology and archaeology
☐	☒ Animals and other organisms
☒	☐ Clinical data
☒	☐ Dual use research of concern

Methods

n/a	Involved in the study
☒	☐ ChIP-seq
☒	☐ Flow cytometry
☒	☐ MRI-based neuroimaging

Antibodies

Antibodies used	Primary antibody: AntiVGLUT2, Synaptic Systems, Cat. No. 135 403, 1:750 dilution.<br>Secondary antibody: Alexa Fluor™ 594, Thermo Fisher Scientific, Cat. No. A-11012, 1:500 dilution.

	We apologize that lot numbers cannot be recovered.
Validation	All relevant information on validation and references provided on the webpage of the company: Primary AB: https://sysy.com/product/135403, Secondary AB: https://www.thermofisher.com/antibody/product/Goat-anti-Rabbit-IgG-H-L-Cross-Adsorbed-Secondary-Antibody-Polyclonal/A-11012

Animals and other research organisms

Policy information about studies involving animals; ARRIVE guidelines recommended for reporting animal research, and Sex and Gender in Research

Laboratory animals	20 male Wistar rats (Harlan Laboratories, San Pietro Al Natisone) were trained/handled on a daily basis and caged in pairs. Data were collected at ages between 6 months to 2.5 years.
Wild animals	No wild animals were used in the study
Reporting on sex	The findings in our study are only limited to male Wistar rats. The housing situation only allowed us to keep animals of one sex to reduce animal stress and improve the rats overall health situation.
Field-collected samples	No field collected samples were used in the study.
Ethics oversight	Approved by Ethics Committee of SISSA and by the Italian Health Ministry (license numbers 569/2015-PR and 570/2015-PR).

Note that full information on the approval of the study protocol must also be provided in the manuscript.
